# [Retracted] Tripterygium glycoside suppresses epithelial-to-­­ mesenchymal transition of diabetic kidney disease podocytes by targeting autophagy through the mTOR/Twist1 pathway

**DOI:** 10.3892/mmr.2026.13935

**Published:** 2026-06-08

**Authors:** Mei Tao, Danna Zheng, Xudong Liang, Diandian Wu, Kang Hu, Juan Jin, Qiang He

Mol Med Rep 24: 592, 2021; DOI: 10.3892/mmr.2021.12231

Following the publication of the above paper, it was drawn to the Editor's attention by a concerned reader that the β-actin control blots featured in Fig. 7C on p. 8 had already appeared previously in an article written by different authors at different research institutes that was also published in the journal *Molecular Medicine Reports*.

Owing to the fact that the contentious data in the above article had already been published prior to its submission to *Molecular Medicine Reports*, the Editor has decided that this paper should be retracted from the Journal. The authors were asked for an explanation to account for these concerns, but the Editorial Office did not receive a reply. The Editor apologizes to the readership for any inconvenience caused.

